# Burnout and patient safety perceptions among surgeons in the United Kingdom during the early phases of the coronavirus disease 2019 pandemic: A two-wave survey

**DOI:** 10.1177/00369330231163378

**Published:** 2023-03-22

**Authors:** Tmam Al-Ghunaim, Judith Johnson, Chandra S Biyani, Marina Yiasemidou, Daryl B O’Connor

**Affiliations:** 1 School of Psychology University of Leeds, Leeds, UK; 2 Department of Urology, St James's University Hospital, Beckett Street, Leeds, West Yorkshire, UK; 3 Bradford Institute for Health Research, Bradford Royal Infirmary, Bradford, UK; 4 School of Public Health and Community Medicine, University of New South Wales, Sydney, Australia; 5 NIHR Academic Clinical Lecturer General Surgery, University of Hull, Hull, UK; 6 ST8 Colorectal Surgery, Bradford Teaching Hospitals, Bradford, UK

**Keywords:** Burnout, coronavirus disease 2019 pandemic, patient safety, surgeons

## Abstract

**Background:**

Surgeons in the UK report high burnout levels. Burnout has been found to be associated with adverse patient outcomes but there are few studies that have examined this association in surgeons and even fewer which have examined this relationship over time.

**Purpose:**

The main aim was to examine the relationships between surgeon burnout and surgeons’ perceptions of patient safety cross-sectionally and longitudinally. The secondary aim was to test whether surgeons’ burnout levels varied over the first six months of the coronavirus disease 2019 pandemic.

**Methods:**

This paper reports data from a two-wave survey (first wave from 5 May and 30 June 2020, the second wave 5 January to 30 February 2021). The dataset was divided into a longitudinal group (for surgeons who responded at both the time points) and two cross-sectional groups (for surgeons who responded at a one-time point, but not the other).

**Results:**

The first key finding was that burnout was associated with patient safety outcomes measured at the same time point (Group 1 = 108, *r =* 0.309*,p* < 0.05 and Group 2 = 84, *r = *0.238, *p* < 0.05). Second, burnout predicted poor patients’ safety perceptions over time, and poor patient safety predicted burnout over time (Group 3 = 39, *p* < 0.05). Third, burnout increased between the first and second surveys (*t* = -4.034, *p* < 0.05).

**Conclusion:**

Burnout in surgeons may have serious implications for patient safety. Interventions to support surgeons should be prioritised, and healthcare organisations, surgeons and psychological specialists should collaborate on their development.

## Introduction

Rates of burnout are high in physicians and have increased following the onset of the coronavirus disease 2019 (COVID-19) pandemic. In a United Kingdom (UK) annual training survey of trainee physicians, 44% of the 63,000 respondents reported having high or very high levels of burnout.^1^ Concerningly, studies on surgeons indicate that they experience higher burnout than most other medical specialities.^2,3^

The causes of high stress and burnout in surgeons are varied and include financial insecurity, fear of lawsuits, peer pressure, and emotional trauma due to patient deaths.^4^ For example, one qualitative study found that interpersonal conflict at work, greater demands than resources, the challenge of work-life balance, and the devastating impact of errors and poor patient outcomes contributed to surgeons’ burnout.^5^ In addition, other quantitative studies have reported that the higher levels of burnout among surgeons are associated with several elements such as greater overall working hours,^6,7^ not being involved in decision-making,^8^ and lack of the training.^9^

Several studies have indicated that high stress and burnout in healthcare professionals are associated with the delivery of poorer patient care. A recent systematic review and meta-analysis of physicians suggested that burnt-out physicians were at twice the risk of being involved in a patient safety incident.^10^ This pattern has also been observed in surgeons specifically. In a recent systematic review of studies in surgeons, burnout was associated with a 2.5-fold higher risk of involvement in medical or surgical errors. However, only 14 studies were identified in this review, and the majority were conducted in the United States (nine of the 14 were American, four were European, and one was Chinese).^11^ None of these studies were conducted in the UK, therefore, there is a need for further evidence to understand the strength and generalisability of this relationship in surgeons in the UK.

Furthermore, while there is an burgeoning literature investigating associations between burnout and poorer patient care, the direction of this association is unclear. Even large, robust surveys tend to be cross-sectional, only providing a snapshot of participants at a single time point.^12–16^ Few studies have sought to understand whether high burnout predicts subsequent poorer patient safety, whether poorer patient safety leads to high burnout, or indeed, whether the relationship is bidirectional. This information could help inform where interventions to improve burnout and patient safety should focus. As such, there is a need for a study that tracks surgeons to elucidate the nature of these associations. There is also a need to better understand how burnout has changed in surgeons over the course of the pandemic.

This study aimed to address these gaps. The main aim was to investigate associations between surgeon burnout and patient safety perceptions at the same time-point and over time. The second aim was to examine whether burnout fluctuated across the early phases of the pandemic in individual surgeons. The study aimed to answer three specific research questions:

1. Is burnout associated with patient safety outcomes measured at the same time point?
2. Is the relationship between burnout and patient safety a two-way, reciprocal relationship over time?
  - Does burnout at Wave 1 predict patient safety perception at Wave 2?
  - Do patient safety perceptions at Wave 1 predict burnout at Wave 2?
3. Did burnout fluctuate across the six months between 30 June 2020 and 5 January 2021?

## Methods

### Design

This research used a longitudinal questionnaire design with data collection at two-time points (at baseline, ‘Wave 1’; and again 6 months later, ‘Wave 2’). Questionnaires were made available both online, and as hard copy paper versions to optimise reach.

This paper reported findings from a two-wave survey. The dataset was divided into three groups for analysis: (1) cross-sectional group 1 contained data from surgeons who provided one survey response between 5 May and 30 June 2020, named ‘Group 1’, (2) cross-sectional group 2 contained data from surgeons who provided one survey response between 5 January to 30 February 2021, named ‘Group 2’; (3) the longitudinal group contained data from the participants who responded to both the first cross-sectional survey (5 May and 30 June 2020) and the second cross-sectional survey (5 January to 30 February 2021), named ‘Group 3’. Each group had different participants.

### Participants and recruitment strategy

All practicing UK surgeons were eligible to take part, regardless of their specialty and whether they were trainees or consultants. Anyone outside that group was excluded, including those who were retired. To determine the appropriate number of participants to recruit, a priori power analysis was run for a correlation on G*Power assuming 80% power and setting alpha at .05 with a medium effect size of 0.30.^17^ The G*Power analysis indicated that the minimum sample size needed for the research was 82. Convenience and snowball sampling strategies were used to recruit participants. Using social media outlets (e.g., Twitter), surgeons and surgical trainees working in various surgical specialties across the UK were invited to participate in the survey, and 306 individual surgeons were contacted using an available networking email list. A five-day reminder was sent after the initial invitation.

### Ethical approval

All potential participants were provided with information about the research study. Participants were informed that their information would be stored safely and securely and that they could withdraw their data up to 2 months after completing the survey. This study received ethical approval from the School of Psychology Research Ethics Committee at the University of Leeds on 4 May 2020 (Ref: PSYC-34).

### Measures

The questionnaires were completed by participants in Wave 1 and Wave 2. The same questionnaire was delivered in both waves. The survey comprised three sections: (1) Demographics: Age, gender, and ethnicity, number of years of experience as a practising surgeon and position, and speciality; (2) Surgeon burnout as evaluated by the Oldenburg Burnout Inventory (OLBI),^18^ and well-being by the General Health Questionnaire (GHQ-12); (3) Patient safety, which included a series of questions relating to the safety of their practice.

### Burnout

The OLBI^18^ scale has eight disengagement items and eight exhaustion items. An example of disengagement is “over time, one can become disconnected from this type of work”; an example of exhaustion is “there are days when I feel tired before I arrive at work”. For each of the separate subscales, burnout was classified as: no exhaustion/disengagement = 0–17.59; mild exhaustion = 17.60–21.99; and severe exhaustion = 22–32. For overall burnout: no burnout = 0–35.1, mild burnout = 35.2–43.9, and high burnout = 44–64.^19^ The four-item Likert rating scale included the following options: Strongly Agree (1), Agree (2), Disagree (3), and Strongly Disagree (4). The OLBI score had a high level of internal consistency (Group 1: Cronbach's α = 0.88, Group 2: α = 0.90, Group 3 first wave α = 0.90 and the second wave α = 0.90). Individually, both subscales had good internal consistency (exhaustion, group: 1 α = 0.85; Group 2: α = 0.83; Group 3, first wave: α = 0.87 and second wave: α = 0.88) (disengagement, Group 1: α = 0.73; Group 2: α = 0.80; Group 3, first wave: α = 0.77 and second wave: α = 0.80).

### General health questionnaire

The GHQ-12 is a unidimensional scale of psychological distress that measures symptoms of anxiety, sadness, social dysfunction, and loss of confidence.^20^ Like other studies,^21,22^ the current study used a four-point Likert scale where participants rated the presence of symptoms (“not at all"=0, “same as usual"=0, “more than usual"=1, “far more than usual"=1). Participants were grouped into four categories according to their GHQ-12 scores: asymptomatic (score 0), subclinical symptomatic (scoring 1–3), symptomatic (score 4–6), and very symptomatic (score 7–12). This scale had good internal consistency in the present study (Group 1: α = 0.90; Group 2: α = 0.84; Group 3, first wave: α = 0.90 and second wave: α = 0.85).

### Patient safety and safe practitioner measures

Participants were asked if they had been responsible for any (a) adverse events (AEs) and/or (b) near misses (NMs) in the preceding three months (Yes or No responses). Participants rated the extent to which they felt they could provide safe care, dependent on work-related conditions. The statement was, “My practice is not as safe as it could be due to work-related factors/conditions” and participants responded on a five-point Likert scale ranging from 1 (Strongly disagree) to 5 (Strongly agree). This is known as the ‘Safe Practitioner’ measure^23^ and has been used in nursing studies where the scores were found to converge with other long-term patient safety measures.^23^

### Data analysis

We had two datasets from which we generated three groups—two cross-sectional for the participants who responded during Wave 1 or Wave 2 but not at both time-points (Groups 1 and 2) and one longitudinal (Group 3), for the participants who completed questionnaires at both time-points. For Groups 1 and 2, eligibility and outliers were checked. Six cases from Group 1 and four cases from Group 2 were dropped for having only completed the demographic questions. The data were found to be missing completely at random (MCAR) using Little's MCAR test (Chi-square = 38.330, df = 370, *p* = 0.609 for Group 1 and Chi-square = 31.450, df = 21, *p* = 0.660 for Group 2). There was no missing data in Group 3.

To address Research Question 1 (is burnout associated with patient safety outcomes measured at the same time point?), Spearman's correlations were used to investigate associations between variables as the data were not normally distributed. To address Research Question 2 (do changes in burnout predict changes in patient safety perceptions over time?), residuals were checked and found to be roughly normally distributed, and the data was found to match the requirements of linearity and homogeneity of variance. Multiple regression was used to assess the link between (1) Wave 1 burnout and wave 2 safety perceptions while controlling for Wave 1 safety perceptions, and (2) Wave 1 safety perceptions and wave 2 burnout while controlling for wave 1 burnout. Missing data were replaced with the column mean, as only 4% of data points were missing.^24^

To address research question 3 (did burnout fluctuate across the pandemic?), a paired samples T-test was used to assess whether burnout, GHQ-12, and safe practice fluctuated between the Wave 1- and Wave 2-time points in Group 3. In all analyses, *p* < 0.05 was considered as statistically significant.

## Results

### Descriptive statistics

Cross-sectional Group 1 included 102 participants with a mean age of 42.15 years (SD 10.58), and cross-sectional Group 2 included 84 participants mean age of 43.06 years (SD 10.53). The longitudinal group (Group 3) had 39 participants, with a mean age of 44.10 years (SD = 11.23). See Table 1 for full participant details.

**Table 1.** Surgeons’ characteristics in all groups.

		Cross-sectional 1 (Group 1)	Cross-sectional 2 (Group 2)	Longitudinal study (Group 3)
Total number of respondents		108	84	39
Mean age		42.15 years (range: 31–66 years), SD: 10.58.	43.05 years (Range: 25–70 years), SD: 10.53	44.10 years (Range: 27–65 years) SD: 11.23
Gender	Male	68 (66.7%)	67 (79.8%)	25 (64%)
	Female	19 (18.6%)	17 (20.2%)	14 (36%)
	Other	1 (1%)	0	0
	Did not respond	14 (13.7%)	0	0
Grade	Consultants	62 (57.4%)	47 (56%),	23 (59%)
	Specialty trainees	27 (25%)	29 (34.5%)	11 (28.2%)
	Core surgical trainee	7 (6.4%)	2 (2.4%)	3(7.7%)
	Other	11.2 (10%)	5 (6%)	2 (5.1%)
	Missing	1 (1%)	1 (1.2%).	0
Specialty	Urology	51(50%)	56 (66.6%)	25 (64.1%)
	General surgery	12 (11.8%)	3 (3.6%)	2 (5.1%),
	Obstetrics and Gynaecology	9 (9.9%)	1 (1.2%).	1 (2.6%).
	Neurosurgery	7 (11.8%)	1 (1.2%).	1 (2.6%).
	Oral and Maxillofacial Surgery	6 (5.9%)	2 (2.4%)	1 (2.6%).
	vascular surgery with colorectal	3 (2.9%)		3(8%)
	HPB and Transplant surgery	1 (1%).		
	Plastic surgery	1 (1%).	1 (1.2%).	1 (2.6%).
	Transplant and hepatobiliary surgery	1 (1%).	2 (2.4%)	
	Otolaryngologists		7 (8.3%)	2 (5.1%),
	Trauma and orthopaedics		6 (7.1%)	2 (5.1%),
	Cardiothoracic surgery		1 (1.2%).	
	Laparoscopic and upper gastrointestinal surgery		1 (1.2%).	
	Maxillofacial		1 (1.2%).	
	Hepatobiliary surgery		1 (1.2%).	
	Ophthalmology			1 (2.6%).
	Missing	1(1%).	0	0

### Research question 1: Is burnout associated with patient safety outcomes measured at the same time point?

#### Group 1

There was a significant positive relationship between burnout and safe practice perceptions *(r = *0*.*309*, p = *0*.*001), and between GHQ and safe practice perceptions (*r* =0 .217, *p* = 0.021) indicating that when burnout was higher, safety perceptions were poorer. In addition, a significant positive relationship was found between higher burnout and a higher risk of reporting a NM (*r* = 0.221, *p* = 0.020), and a significant positive relationship between higher GHQ score and greater risk of a NM (*r* =0 .232, *p* = 0.010) see Table 2.

**Table 2.** Spearman's correlations in group 1 (*n* = 102*)*.

	(1) Burnout	(2) General Health Questionnaire (GHQ-12)	(3) Adverse event/s	(4) Near misses	(5) Safe practicing
(1) Burnout	-				
(2) GHQ-12	0.769**	-			
(3) Adverse event/s	−0.039	030	-		
(4) Near misses	0.221*	0.232*	−0.126	-	
(5) Safe practicing	0.309**	0.217*	−0.076	0.311**	-

**Correlation is significant at the 0.01 level (2-tailed).

*Correlation is significant at the 0.05 level (2-tailed).

#### Group 2

In Group 2, there was a significant relationship between burnout and safe practice perceptions (*r = *0.238*, p = *0.021), indicating that burnout and safe practice were related (Table 3). There was also a significant relationship between higher burnout and greater risk of a NM (*r* = 0.228, *p* = 0.030). There was no significant relationship between GHQ and safe practice (*r* = 142, *p* = 0.320).

**Table 3.** Spearman correlation Group 2 (*n* = 84*)*.

	(1) Burnout group 2	(2) General Health Questionnaire (GHQ-12) Group 2	(3) Adverse event 2 months	(4) Near misses Group 2	(5) Safe practicing Group 2
(1) Burnout Group 2	**-**				
(2) GHQ-12 Group 2	0.568**	**-**			
(3)vAdverse event 2 months	−0.015	0.117	**-**		
(4) Near miss 2 months	0.228*	−0.086	−0.043	**-**	
(5) Safe practicing Group2	0.238*	142	0.331**	0.207	**-**

**Correlation is significant at the 0.01 level (2-tailed).

*Correlation is significant at the 0.05 level (2-tailed).

### Research question 2: Is the relationship between burnout and patient safety a two-way, reciprocal relationship?

This question was investigated using the longitudinal group (Group 3) dataset. The question had two parts.

#### Part one: Does burnout at wave 1 predict patient safety perceptions at wave 2

Age and gender were not significantly related to burnout and patient safety perceptions, so they were not included in the multiple regression analysis.

In Step 1 of the analysis, Wave 1 burnout was a significant predictor for Wave 2 safe practice perceptions, *F(1) = *9.028*, p = *0.005, accounting for 19% variance (see Step 1 for the regression model in Table 4). In Step 2, Wave 1 safe practice perceptions explained an additional 8% of the variance in Wave 2 safe practice perceptions, but the effect of burnout remained statistically significant*, F(2) = *6.965*, p = *0.003. Thus, we found that Wave 1 burnout not only predicted poorer Wave 2 safe practice perceptions, but it also predicted poorer safe practice perceptions when controlling for Wave 1 safe practice.

**Table 4.** A hierarchical linear regression model was used to determine whether burnout during Wave 1 predicted patient safety perceptions during Wave 2*.*

Measure	Steps	Variables	B step 1	B step 2	R2 change for step	R-square.
Patient safety perceptions at wave 2	Step 1	Burnout T1	0.066**	0.047*	0.196**	0.196
	Step 2	Safe practicing T1		0.273*	0.083*	0.279
Burnout at wave 2	Step 1	Safe practicing T1	2.164*	0.330	0.143*	0.143
	Step 2	Burnout T1		0.776**	0.522**	0.665

**p < 0.001.

*p < 0.05.

#### Part two: Does patient safety at wave 1 predict burnout perception at wave 2

In Step 1, Wave 1 safe practice perceptions were a significant predictor for Wave 2 burnout, *F(1) = 6.179, P = *0.018 (see Table 4). However, in Step 2, when Wave 1 burnout entered the equation, burnout was a significant predictor *F(2) = 35.716, P.000*, accounting for 52% variance, but safe practice perceptions were no longer significant *B* = 0.330, *P* = 0.588(see table 4) Thus, we observed that the effect of Wave 1 safe practicing perceptions predicted burnout at Wave 2, but not when controlling for Wave 1 burnout.

#### Research question 3: Did burnout fluctuate across the pandemic?

Compared with Wave 1 *(*mean* = *35.487, SD* = *7.556), Wave 2 burnout scores (mean* = *38.404, SD* = *7.417) were significantly higher (*t = *-4.034, *p < *0.005 (−4.435–1.471) indicating an increase in burnout over time.

## Discussion

Three main findings emerged from the current study. First, burnout was associated with poorer patient safety perceptions when they were measured at the same time point. Second, higher burnout predicted poorer patient safety perceptions over time. Third, burnout significantly increased over the 6 months between 30 June 2020 and 30 February 2021. These findings advance the literature in a number of important ways. First, they provide further evidence that burnout is consistently associated with patient safety perceptions in surgeons. Second, they indicate that the relationship between burnout and safety perceptions may be bi-directional, but that the influence of burnout on safety perceptions may be stronger than vice-versa. Third, they provide further evidence indicating an increase of burnout in surgeons following the onset of the pandemic.

There have been a number of studies investigating cross-sectional associations between burnout and patient safety in healthcare professionals,^12–15^ but few have looked at associations over time. A study by West et al. (2006) which aimed to determine the association between self-perceived medical errors and burnout among physicians in the US. The study found self-perceived medical errors were associated with high burnout. Furthermore higher burnout in all domains was connected with an increase in the probabilities of self-perceived inaccuracy in the following three months.^25^ This is in line with the present study which found evidence of a bi-directional association between burnout and safety perceptions, and a cross-sectional association between burnout and NMs but not with adverse events. There may have been fewer operations performed during the time of this study as a result of the Department of Health's request that surgeons postpone routine procedures during the first Wave of the COVID-19 pandemic.^26^ This may have contributed to a reduction in adverse events.

Our finding that burnout increased over six months during the early phases of the pandemic is in line with a survey of physicians in Japan by Kannampallil et al.^27^ Their study found that 46.3% were experiencing high burnout before the COVID-19 pandemic with a further increase of 12% following the start of the COVID-19 pandemic. Furthermore, large national surveys have also begun to reveal evidence of increasing burnout. For example, the UK training survey,^1^ which included 63,000 trainee doctors found that 56% of them were suffering from high or very high levels of burnout, compared with 43% in early 2020 (GMC, 2021). The current study contributes to the existing literature by following participants during the first wave of COVID-19 and again 6 months later in early 2021, so making our estimations more accurate and less susceptible to bias.

## Implications

These findings emphasise the importance of providing effective burnout interventions in order to improve patient care. There are now local and national support systems in place, such as mindfulness programmes or the Confidential Support and Advice Service of the Royal College of Surgeons.^28^ However, there is a need to increase awareness of the causes and effects of burnout in surgeons, as there are some surgeons who neither recognise nor understand burnout.^29^ Therefore, there is a need to increase the awareness of burnout surgeons and reducing the stigma of asking for mental health help. Also these treatments can be broadly disseminated to all trainees as a preventive measure, lowering the likelihood that trainees decide not to engage, owing to the stigma associated with reporting burnout.^7^ A promising new intervention called Reboot (REcovery BOOsTing coaching) focuses on assisting healthcare professionals with emotionally taxing clinical difficulties, such as adverse events and patient safety incidents, that organisational cannot manage.^30^ When the Reboot programme was applied recently, it helped reduce burnout and depressive symptoms. However, this intervention was delivered on nurses^31^ and no study has yet examined the possible usefulness of Reboot in surgeons. Moreover, there is a growing evidence-base that has shown that group-based acceptance and commitment therapy interventions are effective in reducing general distress in healthcare professionals.^22^

## Strengths and limitations

This study has several strengths. First, it collected data using a two-wave approach. Second, it measured the relationship over time to provide evidence of longitudinal associations. However, some limitations should be considered when interpreting the results, including a relatively small sample size, although it should be noted that we met minimum sample size recommendations.^32^ The findings of this study and other research indicate how crucial it is for surgical trainees to continue receiving support by resources, guidance, and psychological care after the COVID-19 pandemic. Future studies, including a larger sample of junior surgeons, are necessary to comprehend causality, track trends, and promote the adoption of support and guidance at the national and local levels to protect the mental health of our future surgeons.^33,34^

## Conclusion

In conclusion, this study provides evidence regarding the reliability of the association between surgeon burnout and patient safety and elucidates that the relationship between these variables appears to be bidirectional. Also, this study highlights the significant stress surgeons suffered during the first wave of the pandemic and the apparent increase in burnout. There is an urgent need for workplace support and mental health interventions to help surgeons deal with the challenges they face. Together, healthcare organisations, surgeons, and psychologists should offer more and better interventions to support surgeons.
